# Malignant Lymphoma in the Psoas Major Muscle

**DOI:** 10.1155/2017/3902748

**Published:** 2017-02-20

**Authors:** Nobuhiro Akuzawa, Takashi Hatori, Aya Takase, Jun Aoki, Shinji Sakurai, Masahiko Kurabayashi

**Affiliations:** ^1^Department of General Medicine, National Hospital Organization Shibukawa Medical Center, 383 Shiroi, Shibukawa, Gunma 377-0280, Japan; ^2^Department of Internal Medicine, Japan Community Health Care Organization Gunma Chuo Hospital, 1-7-13 Koun-cho, Maebashi, Gunma 371-0025, Japan; ^3^Department of Radiology, Japan Community Health Care Organization Gunma Chuo Hospital, 1-7-13 Koun-cho, Maebashi, Gunma 371-0025, Japan; ^4^Department of Pathology, Japan Community Health Care Organization Gunma Chuo Hospital, 1-7-13 Koun-cho, Maebashi, Gunma 371-0025, Japan; ^5^Department of Medicine and Biological Science, Gunma University Graduate School of Medicine, 3-39-22 Showa-machi, Maebashi, Gunma 371-8511, Japan

## Abstract

An 84-year-old Japanese man taking warfarin to prevent cerebral infarction secondary to atrial fibrillation was admitted to our hospital for evaluation of a painless right back mass. Magnetic resonance imaging (MRI) showed an oval-shaped mass in the right psoas major muscle. The mass showed high intensity on T1-, T2-, and diffusion-weighted imaging and mimicked an acute-phase hematoma. However, it showed no chronological changes typical of a hematoma, and MRI revealed enlargement of the mass 1 week after admission. Histopathological examination of a biopsy specimen revealed diffuse large B-cell lymphoma (DLBCL). Although skeletal muscle lymphoma is rare, physicians should be familiar with its MRI characteristics. In addition, determination of the lymphoma subtype has important implications for the treatment of skeletal muscle lymphoma because DLCBL may have an especially poor prognosis.

## 1. Introduction

Non-Hodgkin lymphoma (NHL) may develop in any extranodal site containing lymphoid tissue, but development in the soft tissue, including the skeletal musculature, is very rare [1]. A recent study showed that extranodal NHL in the soft tissue constitutes 0.2% of all lymphomas [2]. Diffuse large B-cell lymphoma (DLBCL) accounts for >50% of soft tissue NHL, and soft tissue DLBCL has a much worse prognosis than indolent B-cell NHL arising from soft tissue [3]. Moreover, soft tissue DLBCL may show aggressive features and a worse prognosis than non-soft tissue DLBCL [3]. In contrast, indolent soft tissue B-cell lymphoma may have a good outcome [3], suggesting the importance of histological diagnosis of soft tissue NHL.

Two types of skeletal muscle lymphoma have been identified: primary extranodal intramuscular lymphoma and intramuscular development of disseminated disease despite the fact that the lymphoma arises in the musculature [4]. B-cell lymphoma is the most common type of skeletal muscle lymphoma; natural killer lymphoma, T-cell lymphoma, and plasmacytoma can also occur in the skeletal musculature [4]. Skeletal muscle lymphoma tends to be intermediate- or high-grade [4].

We herein report a rare case of an adult who presented with an indolent right back tumor as a result of NHL (DLBCL) arising from the right psoas major muscle. The tumor showed low intensity on T1- and slightly high intensity on T2-weighted magnetic resonance imaging (MRI) and mimicked a hematoma. However, this tumor did not show the chronological changes typical of a hematoma on follow-up MRI, and a computed tomography- (CT-) guided biopsy was needed for a definitive diagnosis.

## 2. Case Presentation

An 84-year-old Japanese man was admitted to our hospital for a detailed examination of an indolent swelling on his right back. He had noticed the swelling on the day of admission; its onset was unknown. He had a 9-year history of treatment with candesartan (4 mg/day), furosemide (20 mg/day), and warfarin (2 mg/day) for hypertension, chronic kidney disease, and atrial fibrillation, but he had no other remarkable medical history or family history. He was a nonsmoker and did not consume alcohol. Prior to admission, he had never presented with pain, fever, or night sweats.

**Figure 1:**
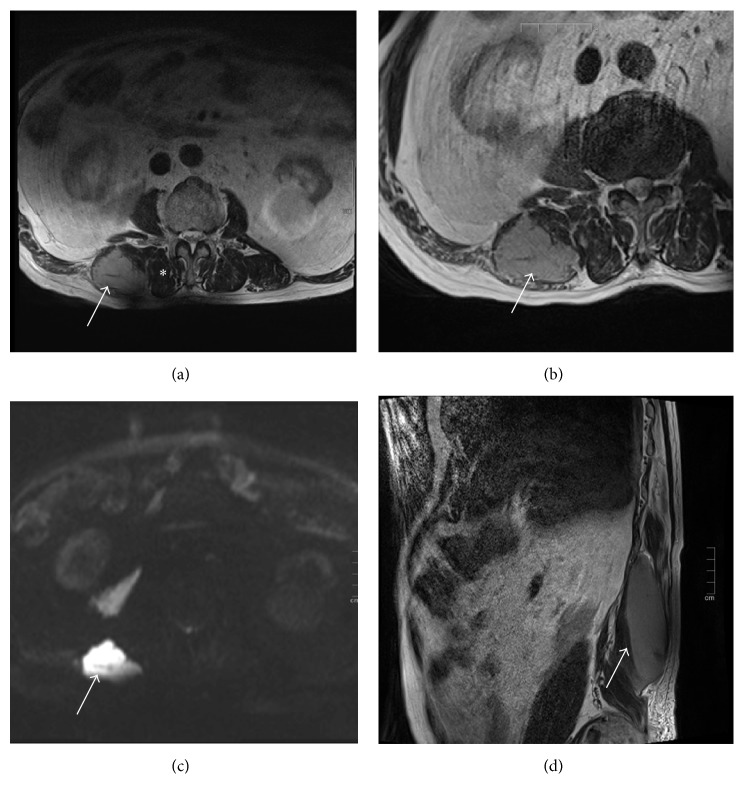
Plain magnetic resonance images of the abdomen on admission. (a) Plain axial T1-weighted image of the abdomen revealed a mass in the right psoas muscle. The mass exhibited higher intensity (white arrow) than the adjacent normal musculature (asterisk). (b) The mass also showed mildly higher intensity on T2-weighted imaging than the adjacent normal musculature (white arrow). (c) The mass showed markedly high intensity on diffusion-weighted imaging (white arrow). (d) Plain sagittal T2-weighted image of the abdomen revealed an oval-shaped mass with homogenous, mildly high intensity inside the right psoas major muscle (white arrow).

On admission, his height was 159 cm, weight was 71 kg, body temperature was 36.8°C, and blood pressure was 132/76 mmHg. His heart rate was 66 beats/min and irregular. Physical examination revealed no significant abnormalities except for a painless elastic hard subcutaneous lump adjacent to the right side of the lumbar vertebrae. The lump was oval in shape and vertically long, measuring about 3 × 10 cm. His arterial blood oxygen saturation was normal at 98%. Both chest and abdominal radiographs showed normal findings. An electrocardiogram showed atrial fibrillation but no other abnormalities. Laboratory tests showed a low blood hemoglobin concentration of 12.7 g/dl (reference range, 13.5–18.0 g/dl), high international normalized ratio-prothrombin time of 1.73 (reference range, 0.85–1.15), high serum lactate dehydrogenase concentration of 270 U/L (reference range, 120–230 U/L), high C-reactive protein concentration of 0.66 mg/dl (reference range, 0.00–0.30 mg/dl), high blood urea nitrogen concentration of 34.8 mg/dl (reference range, 8.0–22.0 mg/dl), and high creatinine concentration of 2.35 mg/dl (reference range, 0.60–1.10 mg/dl). Because his renal dysfunction imposed a restriction upon the use of contrast agent, plain 3 T MRI of the abdomen, including the lumbar region, was performed. MRI of the abdomen revealed an oval-shaped lesion in the right psoas major muscle measuring 4 × 11 cm and exhibiting homogenous internal intensity (Figure 1). This mass showed mildly high intensity on both T1- and T2-weighted imaging compared with the adjacent muscle, but its intensity on T2-weighted imaging was lower than that of water. The mass also showed high intensity on diffusion-weighted imaging, corresponding to the characteristics of an acute-phase hematoma, although the patient had no history of trauma that might have caused an intramuscular hematoma. Based on these findings, the oral warfarin administration was temporarily stopped on day 2 after providing the patient with information regarding the increased risk of cerebral infarction and obtaining his consent.

**Figure 2:**
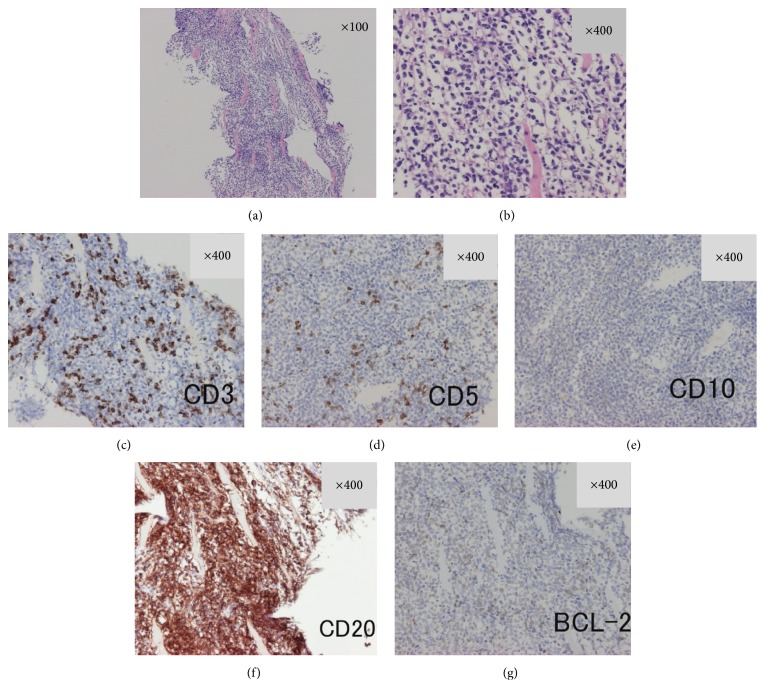
Histopathological examination of a biopsy specimen from the right back mass. (a) Low-power view (×100) and (b) high-power view (×400) of the biopsy specimen obtained from the right back mass, stained with hematoxylin and eosin, showed diffuse infiltration of medium-sized atypical lymphocytes into the interstitium of the striated muscle. Immunohistochemical staining with (c) anti-CD3, (d) anti-CD5, (e) anti-CD10, (f) anti-CD20, and (g) anti-BCL-2 antibodies. CD3- and CD5-positive cells were interspersed among atypical lymphocytes, but CD20-positive cells were obviously predominant. These atypical lymphocytes were CD10-negative and weakly BCL-2-positive.

The patient underwent follow-up MRI on day 7 to ascertain whether this oval-shaped mass had changed with time, which is typically observed in hematomas. However, the internal intensity of the lesion was almost identical to that on admission, and the size of the lesion had slightly increased. Accordingly, a CT-guided needle biopsy was performed on day 9. Blood was not aspirated during the biopsy procedure, and a solid tissue specimen was obtained. Histopathological examination revealed diffuse infiltration of large, atypical cells with irregularly shaped nuclei among the striate muscle fibers (Figure 2). These atypical cells lacked abnormal lymphoid follicle formation and were predominantly CD20-positive on immunostaining. These cells were also weakly BCL-2–positive but CD3-, CD5-, and CD10-negative. The pathological diagnosis was DLBCL. Plain CT of the neck, chest, and abdomen on day 15 showed no significant lymphadenopathy. We consulted with the hematologists of our institution regarding curative treatment, but they discouraged immunochemotherapy because of the patient's renal dysfunction. We proposed additional examinations including detailed analysis of the lymphoma tissue using flow cytometry and ^18^F-fluorodeoxyglucose positron emission tomography (F-FDG-PET), but the patient refused. He was discharged from our hospital on day 16 and lost to follow-up.

## 3. Discussion

In the present case, the MRI characteristics of the mass were similar to those of an acute-phase hematoma observed in patients with intracranial hemorrhage [5]. Specifically, immediately after development of intracranial hemorrhage (hyperacute phase), such hematomas are isointense on T1-weighted imaging and isointense to hyperintense on T2-weighted imaging compared with the surrounding brain tissue, and the T2 signal intensity of the hematoma gradually decreases 1 to 2 days after the hemorrhage [5]. The T1 signal gradually and temporarily increases in the subacute phase, but eventually becomes isointense on T1- and hyperintense on T2-weighted imaging in the center of the hematoma in the chronic phase [5]. In the present case, the MRI findings of the mass in the psoas major muscle on admission were consistent with the characteristics of a relatively new hematoma, and this delayed the definitive diagnosis. Onset of a spontaneous iliopsoas hematoma is rare and may be associated with anticoagulation therapy, but it is a life-threatening condition with a high mortality rate of 30% [6]. This fact contrasts with the stable condition of our patient. A recent study involving a rat model also showed that intramuscular hematomas may show persistent high intensity on both T1- and T2-weighted imaging from the acute stage onward [7], suggesting the importance of accurate image interpretation. Skeletal muscle lymphoma may be hyperintense or isointense compared with the unaffected musculature on T1-weighted images; it also shows homogenous enhancement in most cases on contrast-enhanced T1-weighted images [4, 8]. In addition, intermediately increased signal intensity compared with fat on T2-weighted images is commonly seen [4, 8]. Notably, the lymphoma lesion in the present case showed markedly high intensity on diffusion-weighted imaging, reflecting the high cellularity and high nucleus-to-cytoplasm ratio of lymphomas and leading to restricted diffusion [9]. Recent studies have indicated the importance of diffusion-weighted imaging for lymphoma staging as well as functional and quantitative evaluation of lymphoma lesions after treatment [9, 10]. Giraudo et al. [11] reported that the combined use of F-FDG-PET and MRI, including diffusion-weighted imaging, showed higher diagnostic sensitivity than did the combined use of F-FDG-PET and CT in patients with lymphoma. Accordingly, physicians should know the importance of diffusion-weighted imaging even for an initial diagnosis of lymphoma. Another important MRI feature of skeletal muscle lymphoma is the lack of peritumoral edema, which may be observed in fewer than half of patients with skeletal muscle lymphoma [4]. Moreover, skeletal muscle lymphoma often shows long segmental lesions with orientation of the tumor along the muscle fascicles as well as intact traversing vessels within the lymphoma tissue [8]. These specific MRI findings of skeletal muscle lymphoma may be of value in the differential diagnosis before biopsy.

Skeletal muscle lymphoma comprises various subtypes. Chun et al. [8] reported that diffuse large B-cell lymphoma, peripheral T-cell lymphoma, natural killer T-cell lymphoma (nasal type), and Burkitt lymphoma were observed in patients with primary skeletal muscle lymphoma. Another study showed that follicular lymphoma and anaplastic large T-cell lymphoma may be present in patients with primary skeletal muscle lymphoma [3]. To the best of our knowledge, however, no studies have analyzed the prognosis of skeletal muscle lymphoma. Derenzini et al. [3] evaluated the prognosis of soft tissue NHL and indicated that DLBCL, which is the most common histological type (>50% of cases) of soft tissue lymphomas, showed a worse 5-year progression-free survival rate (34%) than indolent B-cell NHL (64%). They also indicated that the prognosis of soft tissue DLBCL may be worse than the average outcome of patients with DLBCL who underwent standard chemotherapy, suggesting the necessity of first-line therapy intensification including autologous stem cell transplantation for treatment of soft tissue DLBCL [3]. DLBCL is commonly classified into two major biologically distinct molecular subtypes: germinal center B-cell-like (GCB) and activated B-cell-like (ABC). Of the two subtypes, GCB DLBCL is associated with better outcomes when treated with standard chemotherapy [12]. GCB DLBCL is characterized by genetic translocation t(14;18)(q32;q21) or immunohistochemical staining showing CD10 positivity or both CD10 and MUM1 negativity [13]. To the best of our knowledge, the proportion of GCB DLBCL and ABC DLBCL among all soft tissue lymphomas has not been reported, but the poor prognosis of soft tissue DLBCL may suggest a high ratio of patients with ABC DLBCL. Actually, past studies have revealed that most testicular DLBCLs do not show the characteristics of GCB DLBCL [14].

A limitation of the present report is our inability to investigate more specific cell surface markers for determination of lymphoma subtypes by immunohistostaining or flow cytometry. This limitation was a result of the small stock of antibodies in our hospital and unavailability of additional biopsy specimens for flow cytometry and genetic analysis. Accordingly, it is difficult to speculate whether the DLBCL in our patient was derived from transformation of preexisting indolent lymphoma (i.e., secondary DLBCL). Likewise, it is difficult to determine whether this patient's DLBCL belonged to the GCB or ABC subtype. However, it is of cardinal importance to distinguish DLBCL from indolent B-cell NHL when diagnosing soft tissue lymphoma because determination of the lymphoma subtype is pivotal to ensuring optimal treatment.

In conclusion, we have presented a rare case of skeletal muscle lymphoma mimicking intramuscular hemorrhage in the right psoas major muscle. The lymphoma cells showed characteristics of the B-cell lineage, and the tumor was diagnosed as DLBCL. Correct interpretation of MRI findings is important for an initial diagnosis of skeletal muscle lymphoma, and determination of the lymphoma subtype has important implications for the treatment of skeletal muscle lymphoma because DLCBL may have an especially poor prognosis.
